# Human coronaviruses and other respiratory infections in young adults on a university campus: Prevalence, symptoms, and shedding

**DOI:** 10.1111/irv.12563

**Published:** 2018-07-24

**Authors:** Brian M. Davis, Betsy Foxman, Arnold S. Monto, Ralph S. Baric, Emily T. Martin, Amra Uzicanin, Jeanette J. Rainey, Allison E. Aiello

**Affiliations:** ^1^ Department of Epidemiology University of Michigan School of Public Health Ann Arbor MI USA; ^2^ Department of Epidemiology Gillings School of Global Public Health Chapel Hill NC USA; ^3^ Division of Global Migration and Quarantine Centers for Disease Control and Prevention Atlanta GA USA; ^4^ Division Global Health Protection Centers for Disease Control and Prevention Atlanta GA USA

**Keywords:** acute respiratory infection, coronavirus, human, influenza, symptoms, university

## Abstract

**Background:**

The prevalence, symptom course, and shedding in persons infected with the 4 most common human coronaviruses (HCoV)‐229E, HKU1, NL63, and OC43 are poorly described.

**Objectives:**

We estimate their prevalence and associated symptoms among college students identified via a social network study design.

**Patients/Methods:**

We collected 1‐3 samples (n = 250 specimens) from 176 participants between October 2012 and January 17, 2013: participants with acute respiratory infection (ARI; cough and body aches or chills or fever/feverishness) and their social contacts. Virus was detected using RT‐PCR.

**Results:**

30.4% (76/250) of specimens tested positive for any virus tested, and 4.8% (12/250) were positive for 2 or more viruses. Human coronaviruses (HCoVs [22.0%; 55/250]), rhinovirus (7.6%; 19/250), and influenza A (6.4%; 16/250) were most prevalent. Symptoms changed significantly over time among ARI participants with HCoV: the prevalence of cough and chills decreased over 6 days (*P* = .04, and *P* = .01, respectively), while runny nose increased over the same period (*P* = .02). HCoV‐NL63 was the most frequent virus detected 6 days following symptom onset (8.9%), followed by rhinovirus (6.7%).

**Conclusions:**

During a 3‐month period covering a single season, HCoVs were common, even among social contacts without respiratory symptoms; specific symptoms may change over the course of HCoV‐associated illness and were similar to symptoms from influenza and rhinovirus.

## 1 INTRODUCTION

As demonstrated by the 2012 discovery of the Middle East Respiratory Syndrome coronavirus (MERS‐CoV) in Saudi Arabia,^1^ human coronaviruses continue to emerge and may become significant public health problems. MERS‐CoV followed closely on the 2003 identification of severe acute respiratory syndrome coronavirus (SARS‐CoV).^2^ Both viruses originated from animal reservoirs and cause significant mortality.^2^, ^3^, ^4^ By contrast, 4 other human coronaviruses (HCoVs), 229E, HKU1, NL63, and OC43, already circulate globally, but generally have low fatality rates.^5^, ^6^, ^7^, ^8^, ^9^, ^10^ These 4 HCoVs also are believed to be derived from zoonotic sources, including bats (NL63, 229E), dromedary camels (299E), or cattle (OC43), although the origins of HKU1 remain uncertain.^11^, ^12^, ^13^, ^14^

The 4 HCoVs are linked to common cold symptoms,^9^, ^10^, ^15^, ^16^ while HCoV‐HKU1 has less definitively been linked to gastrointestinal symptoms.^17^, ^18^ HCoV‐HKU1 and HCoV‐NL63 can cause severe diseases, including bronchitis, bronchiolitis, and/or croup among pediatric and adult hospitalized patients.^5^, ^7^, ^8^, ^19^, ^20^, ^21^ However, due to the relatively mild course of illness in the majority of otherwise healthy individuals, these 4 HCoVs are thought to be underreported.^22^

Our current understanding of the epidemiology of HCoV‐229E, HCoV‐HKU1, HCoV‐NL63, and HCoV‐OC43 outside of clinics is extremely limited. The prevalence, severity, and co‐occurrence of HCoVs with other respiratory viruses are not yet established.^4^ Data are primarily from outbreak reports, case studies, and clinical studies focusing predominantly on children.^5^, ^6^, ^8^, ^15^, ^18^ Here, we begin to address this gap by estimating the prevalence, shedding duration, symptom progression, and codetection with other respiratory viruses of HCOV‐229E, HKU1, NL63, and OC43 among a cohort of college‐aged students.

## 2 METHODS

We collected demographic, clinical data, and throat and anterior nasal specimens from students as part of a previously described large social network study of acute respiratory infection (ARI) among university students.^23^ Briefly, a total of 590 students living in one of 6 on‐campus residence halls were recruited through a chain referral method between October 2012 and January 17, 2013. All participants were asked to identify recent social contacts through searching a list of enrolled contacts or through suggestions based on the underlying social network on a weekly online survey. For a 10‐week period from January 17 until April 9, 2013, participants experiencing respiratory symptoms were asked to complete an online screening survey to self‐report illness symptoms.

Participants reporting symptoms meeting the ARI case definition (cough plus at least one of the following: body aches, chills, or fever/feverishness) were scheduled to provide up to 3 specimens over a 6‐day period following ARI onset. In order to reduce the likelihood that any 2‐illness episodes were linked to the same etiology, symptom‐onset dates were required to be *at least* 2 weeks apart for an ARI participant to provide more than one set during the study period. This allowed us to consider each illness episode as an independent event.

### 2.1 Social contacts

Once an ARI case was identified through our online screening survey, an email was automatically sent out to the individual's network contacts, inviting presumed “healthy” social contacts to provide a specimen. The social network was identified through a list of contacts that each enrollee generated over the course of the study. Social contacts were eligible if: (i) they had recent face‐to‐face contact within the previous calendar week with an ARI participant and (ii) were not an ARI participant during the previous 2 weeks. Social contacts that elected to provide specimens were scheduled for up to 3 specimen collections.

Although healthy social contacts were not experiencing ARI when they were asked to provide a specimen, some of the social contacts reported symptoms of illness, such as cough or sneezing, at the time of specimen collection. Changes in symptoms among social contacts were calculated as the time from the first specimen collection to illness onset. Any social contact symptomatic on any one or more of the specimen collection days was defined as a “social contact with symptoms.” Any social contact remaining healthy on specimen collection days 0, 3, and 6 was defined as an “asymptomatic social contact.”

The University of Michigan's Institutional Review Board (IRB) (HUM00054432) approved the study protocol, and the Centers for Disease Control and Prevention's Human Subjects Research Office reviewed and approved deferral to the University of Michigan's IRB.

### 2.2 Symptom assessment

All participants providing specimens reported information on 13 acute symptoms: abdominal pain, body aches, chills, cough, diarrhea, earache, feverishness, headache, nasal congestion, runny nose, sneezing, sore throat, and vomiting. Symptoms were collected using a standardized questionnaire administered by trained staff during the sample collection visit, and severity was reported as follows: not present, mild, moderate, or severe.

### 2.3 Specimen collection and testing

For each ARI illness participant and invited social contact, we aimed to collect up to 3 samples from each study participant as follows:

#### 2.3.1 ARI participants

Day 0 specimen—within 24 hours of illness onset.
Day 3 specimen—between 25 and 96 hours after illness onset.
Day 6 specimen—between 97 and 144 hours after illness onset.

#### 2.3.2 Social contacts

Day 0 specimen—time of first specimen collected, as close to illness onset of ARI contact as possible.
Day 3 specimen—approximately 72 hours after initial specimen collection.
Day 6 specimen—approximately 144 hours after initial specimen collection.

If a social contact reported symptoms consistent with our ARI definition, either through the online screening survey or during specimen collection, they were considered an ARI participant and their next scheduled specimen was considered a day 0 ARI specimen. The collection of any combination of day 0, day 3, and day 6 specimens for any participant was defined as a “set” of specimens.

Trained staff collected specimens at each participant's residence. Swabs were taken from 2 locations: the anterior nares and along the uvula. Both specimens were placed in Copan Universal Transport Media (Copan, Murrieta, California) and then stored at −70°C prior to testing.

All specimens were tested for 13 respiratory viruses: coronaviruses 229E, HKU1, NL63, and OC43; adenovirus; human metapneumovirus (hMPV); influenza A and B; parainfluenza 1, 2, and 3; rhinovirus; and respiratory syncytial virus (RSV). For all viruses except influenza A/B, aliquots from the throat and nasal swab were combined prior to testing. Influenza A/B testing was performed separately on throat and nasal swabs, and participants were considered positive for influenza if either swab tested positive.

The number of specimens collected per episode ranged from 1 to 3 per set. For each illness episode, participants and each of their social contacts received an incentive of $15 for their first specimen, $20 for their second, and $25 for their third specimen within a collection period.

Tests for all respiratory viruses were performed in the laboratory using real‐time reverse transcriptase polymerase chain reaction (RT‐PCR). Primers and probes were developed by the Centers for Disease Control and Prevention (CDC) and obtained from the Division of Viral Disease, Gastroenteritis, and Respiratory Viruses and the Influenza Division. Additional information about the RT‐PCR process and RNA/DNA extraction can be found elsewhere.^24^ We assessed the type and number of viral pathogens in each of the day 0, 3, and 6 specimens. A participant was considered positive for a particular virus (or viruses) if at least one of the 3 specimens within an illness episode had a positive RT‐PCR result.

### 2.4 Statistical analysis

We used Fisher's exact tests and t tests to compare demographic differences between study participants providing and not providing specimens, as well as the virus prevalence between 3 groups: (i) ARI participants, (ii) social contacts with symptoms, and (iii) healthy social contacts. Symptoms were analyzed as present or absent, except for cough, which, as a required symptom for the ARI case definition, was defined as absent/mild compared to moderate/severe. To assess changes in symptoms over time, we compared the proportion of participants who reported each symptom on day 0, 3, and 6 for each illness episode, testing for trends by virus with the Cochran‐Armitage test. We assessed the change in illness symptoms over the 6‐day period separately for ARI participants (with a defined symptom‐onset date) and social contacts with symptoms (with no defined symptom‐onset date). Due to sample size constraints, the 4 human coronaviruses were combined for symptom analysis. All statistical analyses were calculated using SAS 10.1 (Cary, NC).

## 3 RESULTS

Of the 590 enrolled participants, 176 (29.8%) provided specimens as an ARI participant, a social contact, or as both an ARI participant and social contact. A total of 250 sets, the collection of 1‐3 specimens over an illness episode, were collected: 81 of 176 (46.0%) participants provided 96 sets of specimens after meeting the ARI case definition; 70 od 176 (39.8%) participants provided 88 sets of specimens as social contacts; and 25 of 176 participants (14.2%) provided 66 sets of specimens (31 sets as an ARI case and 35 sets as social contacts); 115 ARI reports were eligible for specimen collection, of those 96 of 115 (83.5%) provided a specimen. A mean of 1.6 specimens was collected per set. Compared to enrolled students who did not report ARI or did not provide specimens as a social contact, those providing specimens were slightly older (19.5 years vs 19.1 years; *P* = .0006), had parents who were less well educated (*P* = .04), and were less likely to have received a 2011/12 seasonal influenza vaccine (37.7% vs 51.2%; *P* = .01; Table 1).

**Table 1:** Demographic Information for the 590 participants enrolled in the eX‐FLU study

	Participants providing specimens (N = 176)	Participants not providing specimens (N = 414)	*P*‐value
Male	75 (42.6)	160 (41.9)	.87
Age; Mean, SD	19.5 (1.2)	19.1 (0.9)	.0009
Race			
White	110 (64.7)	254 (68.7)	.36
Black	13 (7.7)	34 (9.2)	
Other	47 (27.7)	82 (22.2)	
Parental education			
<College	43 (25.0)	62 (16.7)	.04
College	49 (28.5)	99 (26.6)	
>College	80 (46.5)	211 (56.7)	
Seasonal influenza vaccination 2012‐13	58 (37.7)	104 (51.2)	.01

### 3.1 Virus prevalence

Just over half (127/250; 50.8%) of the specimen sets were from ARI participants, 78 (31.2%) from social contact with symptoms, and 45 (18.0%) from asymptomatic social contacts. Overall, 76 (30.4%) of the 250 sets were positive for at least one of the 13 viruses included in our assay; a total of 101 viruses were identified (11 dual infections, one triple infection). The overall prevalence of virus from ARI participants was 46.5%, compared to 28.3% for social contacts with symptoms (*P* = .01) and 13.3% for asymptomatic social contacts (*P* < .001). The most common virus identified was HCoV‐NL63 (10.0%; 25/250), followed by rhinovirus (7.6%; 19/250), influenza A (6.4%; 16/250), and RSV (3.2%; 8/250). Influenza A was the only virus that appeared statistically significantly more frequent in ARI cases than social contacts with symptoms or asymptomatic social contacts (ARI participants 10.2% vs social contact with symptoms 2.6%, *P* = .05); though not between ARI participants and asymptomatic social contacts 2.2%, *P* = .12). No specimens tested positive for parainfluenza 2 (Table 2).

**Table 2:** Prevalence of RT‐PCR viral detection among 176 participants with 250 specimen sets using symptom status from the eX‐FLU study in the university setting

Identified virus	ARI participant^a^ n = 127 (%)	Social contacts		*P*‐value^b^: ARI vs SC with symptoms	*P*‐value: ARI vs asymptomatic SC
		With symptoms n = 78 (%)	Asymptomatic n = 45		
HCoV‐229E	5 (3.9)	2 (2.6)	1 (2.2)	.71	1.00
HCoV‐HKU1	1 (0.8)	2 (2.6)	0 (0.0)	.56	1.00
HCoV‐NL63	17 (13.4)	6 (7.7)	2 (4.4)	.26	.16
HCoV‐OC43	4 (3.1)	1 (1.3)	0 (0.0)	.65	.57
Influenza A	13 (10.2)	2 (2.6)	1 (2.2)	.05	.12
Influenza B	2 (1.6)	0 (0.0)	0 (0.0)	.53	1.00
Adenovirus	2 (1.6)	1 (1.3)	0 (0.0)	1.00	.00
Human metapneumovirus	4 (3.1)	1 (1.3)	1 (2.2)	.65	1.00
Parainfluenza 1	0 (0.0)	1 (1.3)	0 (0.0)	.38	‐
Parainfluenza 2	0 (0.0)	0 (0.0)	0 (0.0)	‐	‐
Parainfluenza 3	1 (0.8)	4 (5.1)	0 (0.0)	.07	1.00
Respiratory syncytial virus	6 (4.7)	2 (2.6)	0 (0.0)	.71	.34
Rhinovirus	13 (10.2)	4 (5.1)	2 (4.4)	.30	.36
Any detected virus	59 (46.5)	22 (28.2)	6 (13.3)	.01	.00006

a ARI, acute respiratory illness consists of a cough plus at least one of the following: body aches, chills, and feverishness.

b *P*‐value calculated using Fisher's exact test.

### 3.2 Viral Codetection

The overall prevalence of codetection (ie, detection of >1 virus per illness episode) in our population was 4.8% (12/250; Table 3). There were 11 two‐virus codetections and one triple codetection in our population (positive for HCoV‐HKU1, influenza A, and rhinovirus). Rhinovirus occurred most frequently as a codetected agent (8 of 12 specimens; 66.7%), while HCoV‐NL63 was present in 50% of the codetected specimens (6/12). The viral positive counts in any one group were too small to draw conclusions about the statistical associations between codetection and clinical symptoms.

**Table 3:** Frequency of 12 laboratory‐identified codetected viruses within a single specimen among 250 specimen sets collected from the eX‐FLU study in the university setting

Identified virus	Human coronaviruses			Influenza A^a^	Respiratory syncytial virus	Rhinovirus
	229E	NL63	OC43			
HCoV‐229E	‐	2	0	0	0	1
HCoV‐NL63		‐	0	1	1	2
HCoV‐OC43			‐	0	0	1
Influenza A				‐	0	1
Respiratory syncytial virus					‐	2
Rhinovirus						‐

a One specimen tested positive for HCoV‐HKU1, influenza A, and rhinovirus.

### 3.3 Persistence of virus shedding over time

Among ARI participants, the prevalence of all viruses detected decreased from time of symptom onset to follow‐up. Influenza A (16.9%) was the most frequently detected virus on the day of illness onset, followed by HCoV‐NL63 (15.3%). Human coronavirus NL63 was the most frequent virus detected 6 days following illness onset (8.9%), followed by rhinovirus (6.7%). Parainfluenza viruses 1 and 2 were not detected in any specimens collected from ARI participants (Table 4).

**Table 4:** Persistence of virus detection by RT‐PCR among 127 specimen sets from participants with ARI^a^ from the ex‐FLU study in the university setting

Identified virus^b^	Day 0 (n = 59)		Day 3 (n = 98)		Day 6 (n = 90)	
	Viral positive	% Positive	Viral positive	% Positive	Viral positive	% Positive
HCoV‐229E	2	3.4	4	4.1	1	1.1
HCoV‐HKU1	1	1.7	0	0.0	0	0.0
HCoV‐NL63	9	15.3	15	15.3	8	8.9
HCoV‐OC43	2	3.4	2	2.0	3	3.3
Influenza A	10	16.9	10	10.2	3	3.3
Influenza B	2	3.4	1	1.0	1	1.1
Adenovirus	2	3.4	1	1.0	1	1.1
Human metapneumovirus	0	0.0	2	2.0	2	2.2
Parainfluenza 3	0	0.0	1	1.0	1	1.1
Respiratory syncytial virus	3	5.1	4	4.1	3	3.3
Rhinovirus	7	11.9	10	10.2	6	6.7

a ARI, acute respiratory illness is defined as a cough plus at least one additional symptom: body aches, chills, and feverishness.

b No ARI participants tested positive for parainfluenza 1 or parainfluenza 2.

### 3.4 Symptoms present during specimen collection

**Figure 1:**
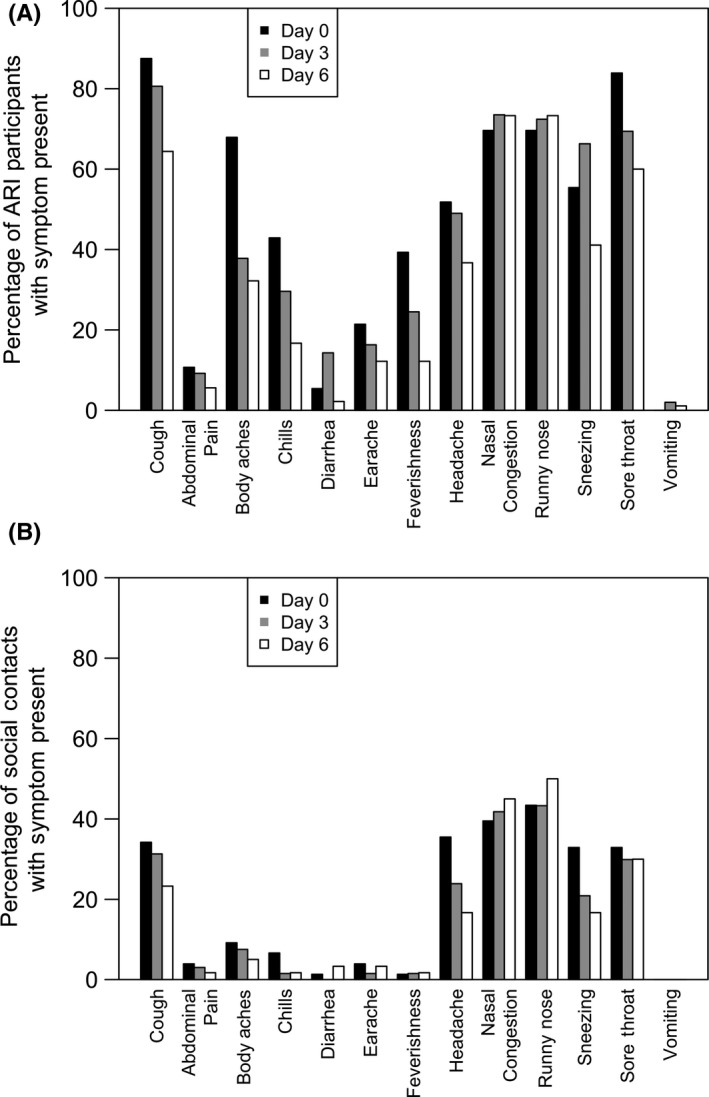
A, Frequency of symptoms present among acute respiratory infection^a^ participants (N = 127) on day 0 (n = 56 specimens), day 3 (n = 98 specimens), and day 6 (n = 90 specimens). B, Frequency of symptoms present among social contacts with symptoms (N = 78) on day 0 (n = 78 specimens), day 3 (n = 67 specimens), and day 6 (n = 60 specimens) following the initial specimen collection^a^. ^a^Cough is defined as moderate or severe vs mild or absent; all other symptoms were either present or absent

Of the 127 participants with ARI, 56 provided a specimen on day 0, 98 provided a specimen on day 3, and 90 provided a specimen on day 6. The most frequent symptoms on day 0 were moderate/severe cough (87.5%) and sore throat (83.9%). By day 3, the most frequent symptoms were moderate/severe cough (80.6%), nasal congestion (73.5%), and runny nose (72.4%). Finally, 6 days following illness onset, the most frequent symptoms were nasal congestion and runny nose (both 73.3%; Figure 1A).

Of the 78 social contacts with symptoms, 78 provided a specimen on day 0, 67 on day 3, and 60 on day 6. The most frequent symptoms across the 6‐day specimen collection time frame were runny nose (43.4% on day 0, 43.3% on day 3, and 50.0% on day 6) and nasal congestion (39.5% on day 0, 41.8% on day 3, and 45.0% on day 6; Figure 1B).

Looking over all the specimens collected in a set, 67.2% (203 of 302 social contact specimens) of specimens collected from social contacts were associated with at least one symptom and 32.5% (98 of 302 social contact specimens) were associated with no symptoms.

### 3.5 Change in symptoms over time

**Figure 2:**
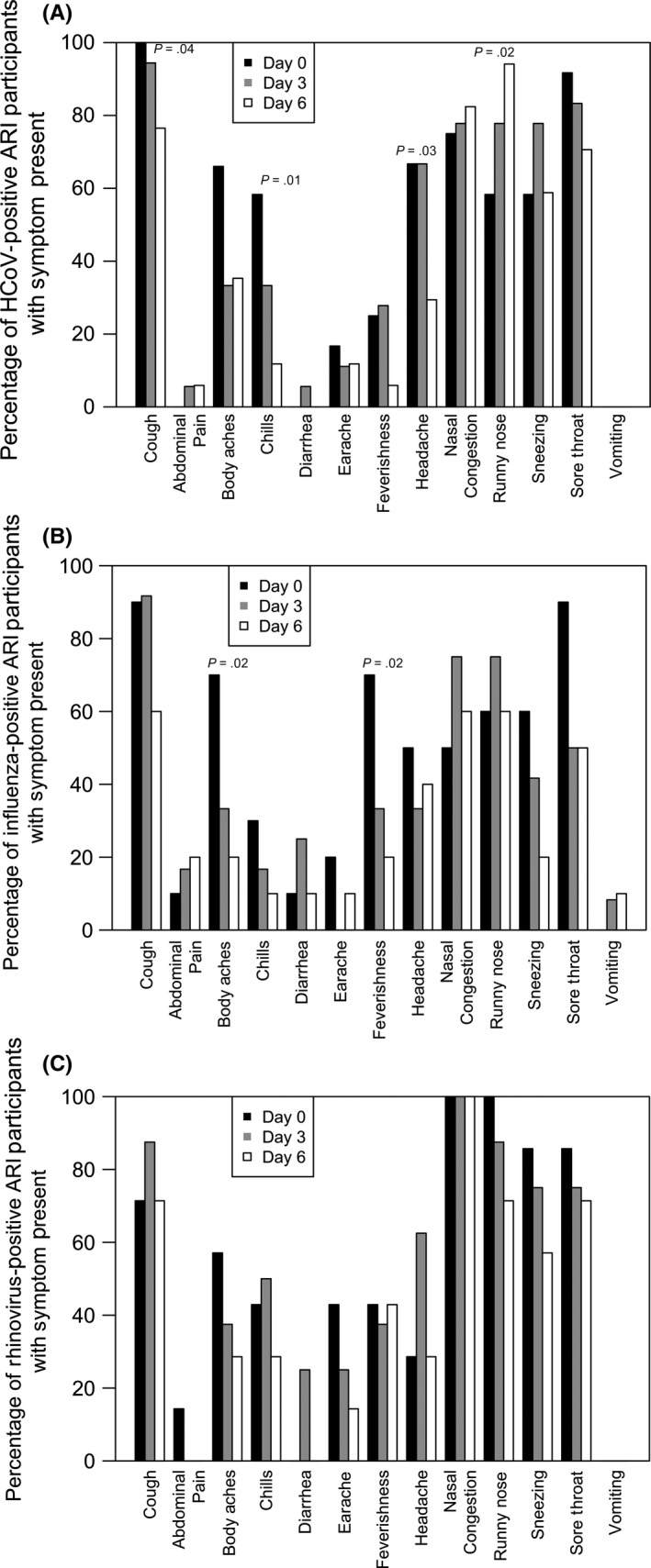
A, Frequency of symptoms present among 19 ARI ^a^ participants positive for at least one of the 4 human coronaviruses on day 0 (n = 12), day 3 (n = 18), and/or day 6 (n = 16) following illness onset^b,c^. B, Frequency of symptoms present among 12 ARI^a^ participants positive for influenza A on day 0 (n = 10), day 3 (n = 12), and/or day 6 (n = 10) following illness onset^b,c^. C, Frequency of symptoms present among 9 ARI^a^ participants positive for rhinovirus on day 0 (n = 7), day 3 (n = 8), and/or day 6 (n = 7) Following illness onset^b^. ^a^ARI, acute respiratory illness defined as a cough plus at least one additional symptom: body aches, chills, and fever/feverishness; ^b^Cough is defined as moderate or severe vs mild or absent; all other symptoms were either present or absent; ^c^ *P*‐values calculated by the Cochran‐Armitage test for trend over the day 0, 3, and 6 specimens

Among ARI participants with HCoV and multiple specimens (n = 19), the most common symptom within 24 hours of symptom onset was moderate/severe cough (12/12; 100%), followed by sore throat (11/12; 91.7%) and nasal congestion (9/12; 75.0%). Three days following symptom onset, moderate/severe cough (17/18; 94.4%) and sore throat (15/18; 83.3%) were the most common symptoms. Six days following symptom onset, the most common symptoms among ARI patients with HCoV were runny nose (16/17; 94.1%) and nasal congestion (14/17; 82.4%). Moderate/severe cough (*P* = .04), chills (*P* = .01), and headache (*P* = .03) decreased in prevalence from day 0 to day 6. Only the reports of rhinitis (*P* = .02) increased over the 6‐day period (Figure 2A).

For ARI patients with influenza A and multiple specimens (n = 12), moderate/severe cough was the most prevalent symptom during the illness episode, followed by sore throat on day 0 and nasal congestion and runny nose on days 3 and 6 of the illness. Body aches (*P* = .02) and feverishness (*P* = .02) were the only symptoms with a significant difference in the prevalence of symptoms over time (Figure 2B).

Among ARI participants with rhinovirus and multiple specimens (n = 9), nasal congestion was present in all participants at all 3 collection times. Runny nose was the second most common symptom, decreasing over the illness period from 100% on day 0 to 71.4% 6 days after symptom onset; there were no significant changes in the prevalence of symptoms over time among ARI participants with rhinovirus (Figure 2C).

**Figure 3:**
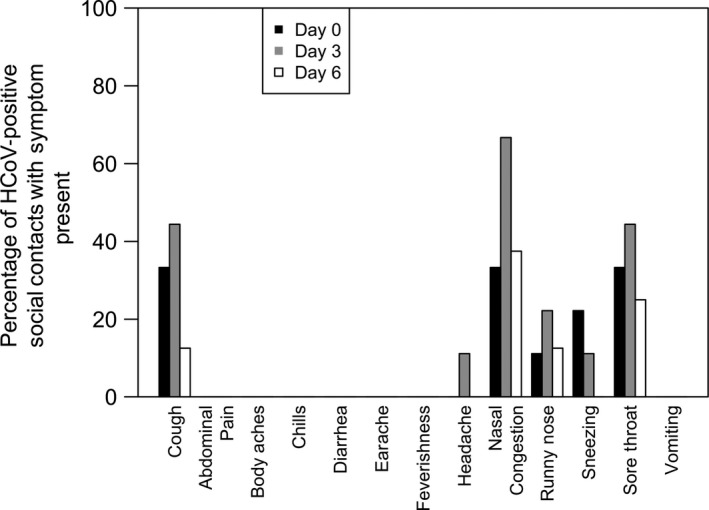
Frequency of symptoms present among 9 social contact participants positive for at least one of the 4 human coronaviruses on day 0 (n = 9), day 3 (n = 9), and/or day 6 (n = 8) following initial specimen collection^a^. ^a^ARI, acute respiratory illness defined as a cough plus at least one additional symptom: body aches, chills, and fever/feverishness; ^b^Cough is defined as moderate or severe vs mild or absent; all other symptoms were either present or absent; ^c^ *P*‐values calculated by the Cochran‐Armitage test for trend over the day 0, 3, and 6 specimens

Symptoms among social contacts were compared at day 0, 3, and 6 for HCoV (n = 9 participants), as this was the most prevalent type of virus identified in this group. Moderate/severe cough, nasal congestion, and sore throat were the most frequent symptoms on day 0 and day 3 of specimen collection. Six days after the initial specimen collection, nasal congestion (37.5%; 3/8) was the most common symptom, followed by sore throat (25%; 2/8) among HCoV‐positive social contacts with symptoms. There were no symptoms with significant changes in the prevalence over time among HCoV‐positive social contacts with symptoms (Figure 3A).

## 4 DISCUSSION

There are few prospective non‐clinic‐based studies describing the epidemiology of human coronaviruses 229E, HKU1, NL63, and OC43 and the changes in symptoms over time. Among the otherwise healthy young adults with ARI symptoms and a sample of their social contacts participating in this study during a single season, winter season, the prevalence of the 4 HCoVs combined was 19.7% among specimens from participants with ARI, 14.1% among social contacts with symptoms, and 6.7% among asymptomatic social contacts. Codetection of viruses was found in 12 specimens collected during the study period, including one triple codetection with HCoV‐HKU1, influenza A, and rhinovirus. Influenza A was the most commonly detected virus among specimens collected from ARI participants, while HCoV‐NL63 was the most frequent virus detected 6 days following illness onset. We found that moderate/severe cough, chills, and headache decreased in frequency over the 6‐day period among students with HCoV infections, while runny nose increased in frequency over the 6‐day period; no similar frequency trends were observed among symptomatic social contacts with HCoV. While statistically significant differences were observed between patients providing specimens and participants not providing specimens in age and parental education, the significantly higher portion of patients not providing specimens with a seasonal influenza vaccination status is likely of concern for interpretation. The differences potentially suggest that receiving a vaccination decreased the likelihood of providing a specimen during our study, an area to note for future studies with a voluntary specimen collection component.

Our prevalence estimates are higher than estimates for a previously conducted study examining these 4 HCoVs in adult and asymptomatic populations, potentially due to the close contact within the residence halls. In addition, our focus on ARI participants and their social contacts did not include individuals living in residence halls that did not have contact with an ARI participant. As such, our reported prevalence estimates among social contacts of ARI cases only are likely higher than they would be among a similar population without known ARI contact. In that retrospective study conducted over 9 years in São Paulo, Brazil, the prevalence of HCoVs tested by RT‐PCR was 8% among 50 adults living in the community with influenza‐like illness.^25^ An additional 50 asymptomatic adults were tested, and no positive HCoV specimens were detected. By contrast, we found that 6.7% of our asymptomatic contacts were positive for HCoVs. A household study that used similar RT‐PCR methods conducted over the same period as our study in southeast Michigan found a prevalence of 16% of HCoVs among individuals with ARI, but they did not examine the prevalence among non‐ARI contacts.^24^

The high prevalence of HCoV, compared to the 12 other viruses in our testing panel, could be attributed to the timing of our study. Human coronaviruses are most frequently found during December through May, and long‐term cohort studies suggest a cyclical pattern in the presence of the 4 HCoVs over multiple years.^26^ However, without multiyear data, we are unable to determine whether the high prevalence of the HCoVs found was due to the cyclical nature of the virus or a result of testing ill individuals in close quarters. Unpublished data from a pilot study conducted among an independent sample of 574 students followed from February to April 2011, resulted in few patients with ARI providing specimens (25), but we found a similar prevalence for HCoVs (16%; 4/25) in a similar young adult population (unpublished data available from corresponding author upon request). Further long‐term annual studies of HCoVs in this community are needed to determine whether there is a seasonal effect or whether there is consistently higher prevalence among young adults in the university setting.

A total of 4.8% (12/250) of specimens were positive with more than one virus, and coronaviruses were found in 44% of the detected codetection. Due to the small sample size, we were unable to assess which characteristics contributed to codetection, including the one individual with 3 detected viruses. Other clinic‐based studies, predominantly among children, have reported the occurrence of codetected viruses.^6^, ^8^, ^27^, ^28^ However, studies outside of the clinical setting are rare. A study of healthy preschool‐aged children in Australia reported twice the prevalence of codetection (56%), but their sample size was smaller (n = 18) and young children tend to have higher rates of respiratory illness than young adults.^29^ These studies suggest that viral codetection is frequent in children. In contrast to these studies, our study designed allowed for multiple samples taken from the same participant, potentially increasing the likelihood that we would find individuals positive for multiple viruses. Overall, coviral infection appears to be less commons among university students compared to younger age individuals. More research is needed on adults to determine risk factors for coinfections among relatively healthy individuals with developed immune systems.

Human coronavirus‐NL63 and rhinovirus had the highest proportion of specimens positive after illness onset. A study examining the viral load of HCoV in children in a daycare setting found an average shedding duration of 6.4 days, with a range of 2.8‐10.1 days,^30^ while a previous rhinovirus challenge study reported patients shedding for at least 4 days, suggesting our findings are not unusual.^31^ However, unlike challenge studies, we were unable to definitely determine the date of infection or adequately sample among patients without symptoms. As such, the interpretation of symptoms over time and detection of virus over time are different for this community‐based study rather than a controlled setting. These findings could influence infection control practices in schools, as well as elsewhere in the community. However, unlike challenge studies, we were unable to definitely determine the precise date of infection or sample every participant without symptoms. As such, the interpretation of symptoms over time and detection of virus over time are different for this community‐based study rather than a controlled setting.

Our findings of persistently high prevalence of runny nose over the 6‐day period in ARI cases with HCoV corresponds with common symptoms found in historical challenge studies of these viruses.^26^ However, we were unable to find any other studies presenting a change in symptoms observed over time for the 4 globally circulating HCoVs outside of human challenge trials. The statistically significant decrease in cough, chills, and headache and increase in runny nose over the 6‐day period for the HCoV observed in our study suggest that symptoms change significantly over the course of natural infection, making it difficult to delineate between viral etiologies associated with common ARI. The similarity of our findings with those of another study conducted in the region during the same season^24^ suggests that university students were under similar regional viral pressure. Due to the low level of severe illness, screening for these viruses in a university setting does not seem necessary. However, it does seem likely that increased testing in the university setting, even among those with mild symptoms, would result in a high number of viruses detected. Future studies would help to confirm the results of this study over multiple seasons to assess long‐term trends that were not observed during the current study, conducted over a single season.

Because we used a chain referral methodology for enrollment, our study population was not randomly recruited. It is unlikely that this would bias the estimates of viral prevalence among those with ARI; however, it is possible that the estimates for viral prevalence from healthy contacts may be elevated compared to the prevalence found in the general population. Additionally, prevalence estimates include samples that were taken at up to 3 time points within the first 6 days of illness, providing a greater opportunity to identify virus‐positive samples compared to other study designs. Further, our testing for viruses was not exhaustive; the 13 viruses included were selected for their frequency of appearance as upper respiratory viruses in the population, as well as their clinical importance. However, additional respiratory viruses may have been present; as a result, the number of codetected viruses identified in this study is likely underestimated. Finally, seasonality may have influenced our findings. By recruiting and testing patients, January‐April of 2012, we were more likely to see respiratory viruses compared to other circulating viruses.^32^

Human coronaviruses are common, even among those without respiratory symptoms, and specific symptoms may change over the course of an illness that can mirror symptoms ranging from influenza to rhinovirus. Larger studies in the community setting are needed to better understand the epidemiological and clinical significance of codetection. These studies may help to uncover important transmission characteristics that could inform measures for addressing more deadly coronavirus outbreaks.

## CONFLICT OF INTEREST

None declared.

## References

[1] de Groot RJ , Baker SC , Baric RS , et al. Middle East respiratory syndrome coronavirus (MERS‐CoV): announcement of the Coronavirus Study Group. J Virol. 2013;87:7790‐7792. 23678167 10.1128/JVI.01244-13 PMC3700179

[2] Drosten C , Gunther S , Preiser W , et al. Identification of a novel coronavirus in patients with severe acute respiratory syndrome. N Engl J Med. 2003;348:1967‐1976. 12690091 10.1056/NEJMoa030747

[3] Breban R , Riou J , Fontanet A . Interhuman transmissibility of Middle East respiratory syndrome coronavirus: estimation of pandemic risk. Lancet. 2013;382:694‐699. 23831141 10.1016/S0140-6736(13)61492-0 PMC7159280

[4] Graham RL , Donaldson EF , Baric RS . A decade after SARS: strategies for controlling emerging coronaviruses. Nat Rev Microbiol. 2013;11:836‐848. 24217413 10.1038/nrmicro3143 PMC5147543

[5] Vabret A , Mourez T , Gouarin S , Petitjean J , Freymuth F . An outbreak of coronavirus OC43 respiratory infection in Normandy, France. Clin Infect Dis. 2003;36:985‐989. 12684910 10.1086/374222 PMC7109673

[6] Vabret A , Mourez T , Dina J , et al. Human coronavirus NL63, France. Emerg Infect Dis. 2005;11:1225‐1229. 16102311 10.3201/eid1108.050110 PMC3320486

[7] Chiu SS , Chan KH , Chu KW , et al. Human coronavirus NL63 infection and other coronavirus infections in children hospitalized with acute respiratory disease in Hong Kong, China. Clin Infect Dis. 2005;40:1721‐1729. 15909257 10.1086/430301 PMC7107956

[8] Kuypers J , Martin ET , Heugel J , Wright N , Morrow R , Englund JA . Clinical disease in children associated with newly described coronavirus subtypes. Pediatrics. 2007;119:e70‐e76. 17130280 10.1542/peds.2006-1406

[9] Tyrrell DA , Bynoe ML . Cultivation of a novel type of common‐cold virus in organ cultures. BMJ. 1965;1:1467‐1470. 14288084 10.1136/bmj.1.5448.1467 PMC2166670

[10] Hamre D , Procknow JJ . A new virus isolated from the human respiratory tract. Proc Soc Exp Biol Med. 1966;121:190‐193. 4285768 10.3181/00379727-121-30734

[11] Huynh J , Li S , Yount B , et al. Evidence supporting a zoonotic origin of human coronavirus strain NL63. J Virol. 2012;86:12816‐12825. 22993147 10.1128/JVI.00906-12 PMC3497669

[12] Pfefferle S , Oppong S , Drexler JF , et al. Distant relatives of severe acute respiratory syndrome coronavirus and close relatives of human coronavirus 229E in bats, Ghana. Emerg Infect Dis. 2009;15:1377‐1384. 19788804 10.3201/eid1509.090224 PMC2819850

[13] Vijgen L , Keyaerts E , Moes E , et al. Complete genomic sequence of human coronavirus OC43: molecular clock analysis suggests a relatively recent zoonotic coronavirus transmission event. J Virol. 2005;79:1595‐1604. 15650185 10.1128/JVI.79.3.1595-1604.2005 PMC544107

[14] Corman VM , Eckerle I , Memish ZA , et al. Link of a ubiquitous human coronavirus to dromedary camels. Proc Natl Acad Sci USA. 2016;113:9864‐9869. 27528677 10.1073/pnas.1604472113 PMC5024591

[15] van der Hoek L , Pyrc K , Jebbink MF , et al. Identification of a new human coronavirus. Nat Med. 2004;10:368‐373. 15034574 10.1038/nm1024 PMC7095789

[16] Woo PC , Lau SK , Chu CM , et al. Characterization and complete genome sequence of a novel coronavirus, coronavirus HKU1, from patients with pneumonia. J Virol. 2005;79:884‐895. 15613317 10.1128/JVI.79.2.884-895.2005 PMC538593

[17] Esper F , Ou Z , Huang YT . Human coronaviruses are uncommon in patients with gastrointestinal illness. J Clin Virol. 2010;48:131‐133. 20362494 10.1016/j.jcv.2010.03.007 PMC2864800

[18] Vabret A , Dina J , Gouarin S , Petitjean J , Corbet S , Freymuth F . Detection of the new human coronavirus HKU1: a report of 6 cases. Clin Infect Dis. 2006;42:634‐639. 16447108 10.1086/500136 PMC7107802

[19] van der Hoek L , Sure K , Ihorst G , et al. Croup is associated with the novel coronavirus NL63. PLoS Med. 2005;2:e240. 16104827 10.1371/journal.pmed.0020240 PMC1188248

[20] Falsey AR , Walsh EE , Hayden FG . Rhinovirus and coronavirus infection‐associated hospitalizations among older adults. J Infect Dis. 2002;185:1338‐1341. 12001053 10.1086/339881 PMC7109881

[21] Talbot HK , Crowe JE Jr , Edwards KM , et al. Coronavirus infection and hospitalizations for acute respiratory illness in young children. J Med Virol. 2009;81:853‐856. 19319948 10.1002/jmv.21443 PMC2767383

[22] Monto AS , Cowling BJ , Peiris JSM . Coronaviruses In: Kaslow RA, Stanberry LR, Le Duc JW, eds. Viral Infections of Humans: Epidemiology and Control, 5 edn New York, NY: Springer; 2014:199‐224.

[23] Aiello AE , Simanek AM , Eisenberg MC , et al. Design and methods of a social network isolation study for reducing respiratory infection transmission: the eX‐FLU cluster randomized trial. Epidemics. 2016;15:38‐55. 27266848 10.1016/j.epidem.2016.01.001 PMC4903923

[24] Monto AS , Malosh RE , Petrie JG , Thompson MG , Ohmit SE . Frequency of acute respiratory illnesses and circulation of respiratory viruses in households with children over 3 surveillance seasons. J Infect Dis. 2014;210:1792‐1799. 24907381 10.1093/infdis/jiu327 PMC4296188

[25] Cabeca TK , Granato C , Bellei N . Epidemiological and clinical features of human coronavirus infections among different subsets of patients. Influenza Other Respir Viruses. 2013;7:1040‐1047. 23462106 10.1111/irv.12101 PMC4634278

[26] Kaslow RA , Stanberry LR , Le Duc JW . Viral Infections of Humans Epidemiology and Control. Boston, MA: Springer US: Imprint: Springer; 2014.

[27] Gaunt ER , Hardie A , Claas EC , Simmonds P , Templeton KE . Epidemiology and clinical presentations of the four human coronaviruses 229E, HKU1, NL63, and OC43 detected over 3 years using a novel multiplex real‐time PCR method. J Clin Microbiol. 2010;48:2940‐2947. 20554810 10.1128/JCM.00636-10 PMC2916580

[28] Esper FP , Spahlinger T , Zhou L . Rate and influence of respiratory virus co‐infection on pandemic (H1N1) influenza disease. J Infect. 2011;63:260‐266. 21546090 10.1016/j.jinf.2011.04.004 PMC3153592

[29] Lambert SB , Allen KM , Druce JD , et al. Community epidemiology of human metapneumovirus, human coronavirus NL63, and other respiratory viruses in healthy preschool‐aged children using parent‐collected specimens. Pediatrics. 2007;120:e929‐e937. 17875651 10.1542/peds.2006-3703

[30] Martin ET , Fairchok MP , Stednick ZJ , Kuypers J , Englund JA . Epidemiology of multiple respiratory viruses in childcare attendees. J Infect Dis. 2013;207:982‐989. 23288925 10.1093/infdis/jis934 PMC7107308

[31] Graham NM , Burrell CJ , Douglas RM , Debelle P , Davies L . Adverse effects of aspirin, acetaminophen, and ibuprofen on immune function, viral shedding, and clinical status in rhinovirus‐infected volunteers. J Infect Dis. 1990;162:1277‐1282. 2172402 10.1093/infdis/162.6.1277

[32] Heikkinen T , Jarvinen A . The common cold. Lancet. 2003;361:51‐59. 12517470 10.1016/S0140-6736(03)12162-9 PMC7112468

